# Number of teeth is associated with facial size in humans

**DOI:** 10.1038/s41598-020-58565-8

**Published:** 2020-02-04

**Authors:** Elias S. Oeschger, Georgios Kanavakis, Demetrios J. Halazonetis, Nikolaos Gkantidis

**Affiliations:** 1 Department of Orthodontics and Dentofacial Orthopedics, University of Bern, CH-3010 Bern, Switzerland; 2 Department of Pediatric Oral Health and Orthodontics, UZB – University School of Dental Medicine, University of Basel, CH-4058 Basel, Switzerland; 3 Department of Orthodontics and Dentofacial orthopedics, Tufts University School of Dental Medicine, Boston, MA USA; 4 Department of Orthodontics, School of Dentistry, National and Kapodistrian University of Athens, GR-11527 Athens, Greece

**Keywords:** Anthropology, Evolutionary theory, Oral anatomy

## Abstract

During human evolution there has been an increase in the size of the brain and the cranium, whereas the size of the face, as well as the size and number of teeth have decreased. In modern humans, the occurrence of missing permanent teeth, namely tooth agenesis, is common. It could be attributed to a biological mechanism of tooth number reduction that has evolved during time and might still be active. Although, if evident, it would add support to this theory, the relationship between this phenotype and craniofacial size remains largely unknown. The present case-control study shows that modern individuals with tooth agenesis have indeed smaller facial configurations. For example, a 15-year-old female with no, one, or ten missing teeth would have a facial centroid size of 511.83, 510.81, or 501.70 mm, respectively. No such effect was observable in the cranial base and the cranium. Our results suggest that common gene regulatory mechanisms that have evolved over time, continue to regulate the number of teeth and facial size of modern humans in a coordinated manner. We anticipate our findings to enrich our understanding of the evolution and development of the human head and kindle future developmental research on this field.

## Introduction

The congenital absence of one or more permanent teeth, known as tooth agenesis, is one of the most common dental anomalies in humans (prevalence: 6.4%)^1^ and is mainly attributed to genetic factors. Other dental anomalies, as well as tooth shape and size, have been associated with tooth agenesis, suggesting a common genetic background for overall development of the dentition^2,3^. Furthermore, several genes related to tooth agenesis in humans have been shown to regulate craniofacial bone morphogenesis^4,5^. Tooth agenesis coexists among other features in more than 150 syndromes; however, it appears more frequently as a sporadic isolated trait or segregates in families^6,7^.

During human evolution the size of the jaws and the face^8^, as well as the size^9^ and the number^10^ of teeth has decreased in response to reduced functional needs^11^. On the contrary the size of the human brain, along with the cranium, has steadily increased^12^. This, as well as the above-mentioned ontogenetic considerations, might suggest the presence of common gene regulatory mechanisms that evolved over time and affect the number of teeth and craniofacial size in a coordinated manner.

The relationship between tooth agenesis and craniofacial morphology has been previously explored, with more recent studies revealing a connection between the two^5,13^. Conflicting results can be attributed to differences in samples and methods. To that extent, it is questionable whether the commonly used conventional cephalometric analysis constitutes an appropriate tool to investigate small differences in craniofacial shape and size^14^. Therefore, alternative approaches, such as geometric morphometrics, have been proposed^15,16^.

Our search revealed no study that has investigated the association between number of teeth and craniofacial size in modern humans. Thus, our aim was to investigate whether there is variation in the size of the craniofacial complex related to tooth agenesis.

## Materials and Methods

The ethical approval of this case-control observational study was provided by the Ethics Commission of the Canton of Bern, Switzerland (Project-ID: 2018-01340) and the Research Committee of the School of Dentistry, National and Kapodistrian University of Athens, Greece (Project-ID: 281, 2/9/2016). The methods were carried out in accordance with the relevant guidelines and regulations. The participants signed an informed consent to allow the use of their data in the study. The study is reported according to the STROBE guidelines.

In order to obtain the study population, orthodontic patient records from the following orthodontic clinics were accessed: a) University of Bern, Switzerland, b) National and Kapodistrian University of Athens, Greece, c) two private practices in Athens and two in Thessaloniki, Greece, and d) one private practice in Biel, Switzerland. Archived consecutive patient files from January 2002 until the end of December 2017 were searched for the identification of eligible subjects.

The inclusion criteria for the tooth agenesis group were the following:

1. Individuals older than 9 years of age and younger than 40 years of age when the pre-treatment radiograph was obtained. In cases younger than 12 years old at the time of the pre-treatment radiograph, radiographs obtained at older ages were also examined to exclude potential presence of late forming teeth, such as the second premolars and the second molars^17,18^. Subjects for which diagnosis could not be confirmed at least at the age of 12 were excluded from the study.
2. White racial background.
3. Individuals with tooth agenesis (congenitally missing teeth), without considering the third molars.
4. No syndromes, systemic diseases, or any other anomalies that affect craniofacial morphology, as reported in the subjects’ medical record.
5. Adequate quality lateral cephalometric radiograph in maximal intercuspation, depicting a reference ruler at the mid-sagittal plane for magnification measurement.
6. Adequate quality panoramic radiographs for identification of missing teeth.
7. No intervention known to influence craniofacial morphology, such as orthodontic treatment, prior to image acquisition.
8. No other severe dental anomaly regarding tooth number, size, or form in any tooth except for third molars.
9. No patient where the reason of absence of any tooth was not definite.

Patient files were reviewed, including the medical and dental history, the intraoral and extraoral photographs, and the radiographs. All relevant data were recorded in an Excel sheet (Microsoft Excel®, Microsoft Corporation, Redmond WA, USA). For each patient, data including sex, race, date of birth, date of image acquisition, panoramic radiograph, cephalometric radiograph, congenitally missing teeth, including third molars, were extracted. The entire sample was reassessed by one researcher (E.S.O.) and any disagreements (intra-rater agreement: 97.5%) were resolved by consensus between him and the last author. The patterns of permanent tooth agenesis in an otherwise normal human population were recorded using the TAC system^19,20^.

Finally, 404 individuals with tooth agenesis (238 females; 166 males), out of more than 8.000 orthodontic patients, fulfilled the inclusion criteria and were selected for the study population. This group is very similar to a group tested in a previous study, where we performed a thorough assessment of non-syndromic permanent tooth agenesis patterns, excluding third molars^20^. The group of that study differed in less than 3% of the cases to the present group, and thus, we do not report a detailed tooth agenesis pattern assessment of the sample here.

The control group comprised individuals without tooth agenesis, except for third molars, and shared all the other inclusion criteria with the study group. For each included subject with agenesis, a control individual matched for age (within 6 months), sex, and geographic origin was included. Thus, a sample of 404 individuals without tooth agenesis (238 females; 166 males) comprised the control group.

In summary, when considering the whole sample (n = 808) and the third molars, 493 individuals had agenesis (mean: 4.1 teeth per subject), whereas when ignoring third molars, 404 individuals had agenesis of other teeth (mean: 2.7 teeth per subject). The frequency distribution of the number of missing teeth in the study sample is provided in Table 1.

**Table 1:** Distribution of total number of missing teeth per individual, with and without including third molars.

	Number of missing teeth	Frequency (percentage) with third molars	Frequency (percentage) without third molars
	0	315 (39.0%)	404 (50.0%)
	1	99 (12.3%)	158 (19.6%)
	2	110 (13.6%)	127 (15.7%)
	3	57 (7.1%)	33 (4.1%)
	4	65 (8.0%)	30 (3.7%)
	5	54 (6.7%)	10 (1.2%)
	6	26 (3.2%)	10 (1.2%)
	7	18 (2.2%)	10 (1.2%)
	8	21 (2.6%)	5 (0.6%)
	9	9 (1.1%)	4 (0.5%)
	10	9 (1.1%)	5 (0.6%)
	11	4 (0.5%)	2 (0.2%)
	12	3 (0.4%)	2 (0.2%)
	13	2 (0.2%)	2 (0.2%)
	14	6 (0.7%)	4 (0.5%)
	15	2 (0.2%)	—
	16	—	1 (0.1%)
	17	2 (0.2%)	—
	18	4 (0.5%)	—
	19	1 (0.1%)	—
	20	—	1 (0.1%)
	24	1 (0.1%)	—
Total	2010/1101*	808 (100%)	808 (100%)

*With/without third molars.

**Figure 1:**
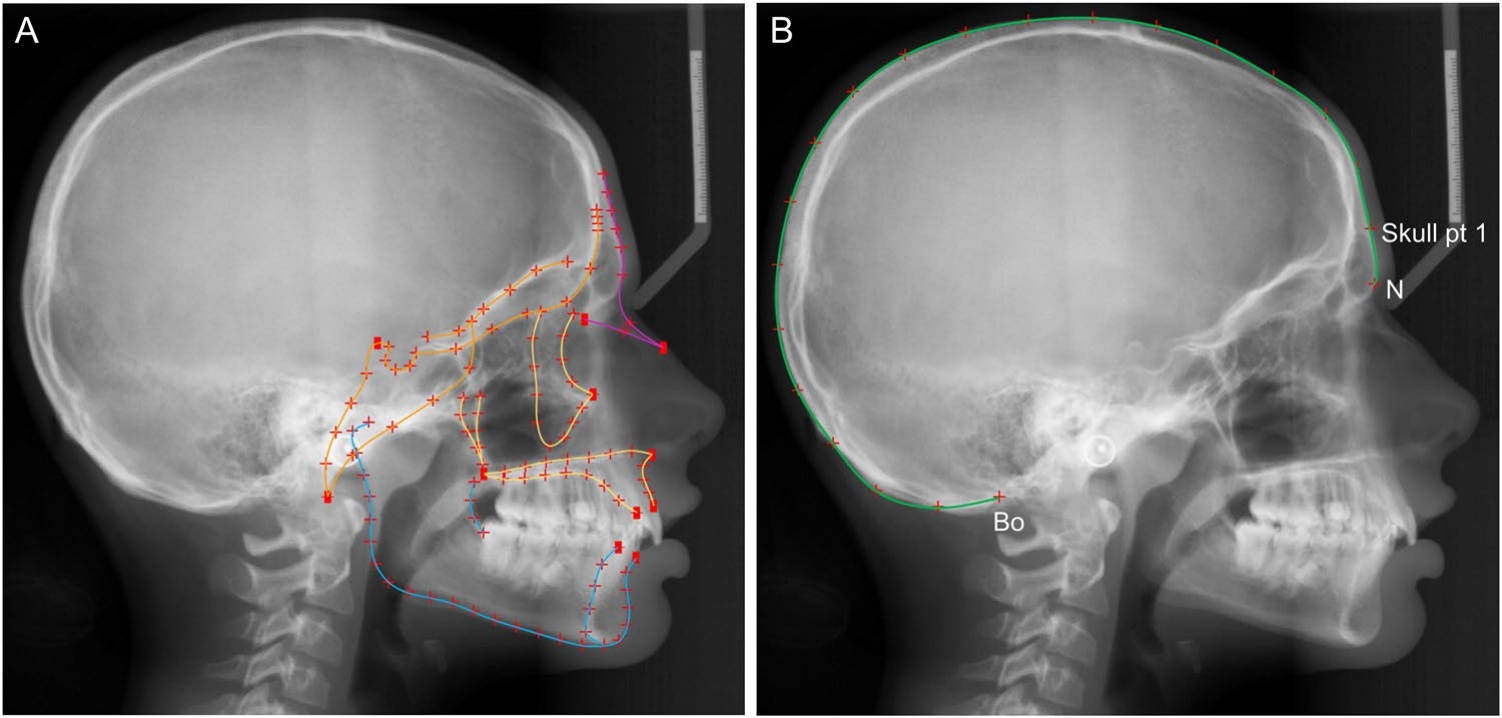
Landmarks used to capture craniofacial morphology. (**A**) Digitization of the craniofacial complex (n = 808) with 15 curves, which included 116 semilandmarks (red crosses), and 11 fixed landmarks (red rectangles). Orange color represents the structures of the cranial base, yellow the maxillary structures, blue the mandibular structures, and all lines together the whole configuration. (**B**) Additional curve digitized in radiographs (n = 112) that also included the whole cranium (green color). This curve started at point N and ended at Bolton (Bo) point. 20 semilandmarks, initially spread equidistantly on this curve were used to capture cranial morphology, starting from a point located at the 5% of the N-Bo distance (Skull pt 1) and ending at Bo point.

Head shape and size information of the agenesis and the control group were captured through landmark identification on the lateral cephalometric radiographs and analyzed through geometric morphometric methods using Viewbox 4 software (dHAL software, Kifissia, Greece). The craniofacial structures were identified and digitally traced with Viewbox 4 software as well, with special configurations that fulfilled the needs of our study^16^. The size of the following anatomical structures was the primary outcome tested in this study: whole craniofacial configuration (not including the superior and posterior part of the cranium), cranial base, maxilla, and mandible (Fig. 1A). Landmarks that would capture the alveolar bone morphology of the jaws were not included, since missing teeth may lead to alveolar bone resorption due to loss of function^21^ and this could have confounded the results. Thus, to avoid local effects due to missing teeth at the anterior area, the four fixed landmarks that were placed in the anterior end of the maxillary and the mandibular bone, on the teeth side, were placed in relation to the cementoenamel junction of the anterior teeth and the overall alveolar bone margin level. The cephalogram size was adjusted to real size using the reference ruler. Fifteen curves comprehensively described the craniofacial skeletal structures, using 127 landmarks, initially distributed equidistantly along these curves. Eleven points identified by local anatomy, such as anterior nasal spine (ANS) and posterior nasal spine (PNS), or positioned at the end of curves, were considered fixed landmarks. All other 116 points were considered semilandmarks^22^ and were thus allowed to slide from their initial position. The sliding of the semilandmarks to minimize bending energy against a reference configuration was performed in an iterative process and an average was computed, which was then used as the reference for the next iteration^23^. This process was repeated three times, until there was no detectable change of the average shape. The subsequent landmark configurations were then superimposed using generalized Procrustes superimposition^24^, which led to the final Procrustes coordinates, that describe the location of each subject in shape space^25^.

Based on the above landmark configurations, size was determined by using the natural logarithm of centroid size (ln(CS))^26^. The centroid size (CS) of a landmark configuration is the square root of the sum of squared distances of a set of landmarks from their centroid^27^. The centroid of a figure is the arithmetic mean position of all the points in the figure. The log transformation of CS was used instead of CS. This is a standard data transformation process used in geometric morphometrics to guarantee that for isotropic landmark variation the distribution in size-and-shape space is isotropic as well and to ensure data normality^16^.

The statistical analysis was conducted with SPSS software (v.20.0, SPSS Inc., U.S.A). In all cases, a two-sided significance test was carried out at an alpha level of 0.05.

Method error was evaluated through repeated digitization and CS measurement of 30 (15 agenesis and 15 control) randomly selected radiographs. No systematic error was detected in any CS variable. The mean difference between repeated ln(CS) measurements was negligible (all points: −0.0014 ± 0.0047, cranial base: −0.0010 ± 0.0097, maxilla: 0.0010 ± 0.0062, mandible: 0.0020 ± 0.0054) as was the mean absolute difference (all points: 0.0034 ± 0.0037, cranial base: 0.0067 ± 0.0070, maxilla: 0.0047 ± 0.0041, mandible: 0.0048 ± 0.0031).

Descriptive statistics and frequency distributions were calculated for sample characteristics and studied variables, respectively.

A multivariate linear regression (general linear model, full factorial) was performed to assess possible associations between the four CS variables (dependent variables: ln(CS) of the craniofacial complex, ln(CS) of the cranial base, ln(CS) of the maxilla, ln(CS) of the mandible) and sex, age, and the number of missing teeth. Following significant results, tests of between-subjects’ effects were performed and parameter estimates were calculated for the regression models. All analyses were performed twice; once including the third molars and once ignoring them.

## Results

When the third molars were considered in the analysis, tests of between-subjects effects showed a significant effect of factors age and sex on all size variables (p < 0.001), whereas the number of missing teeth significantly affected only the maxilla (p < 0.001) and the whole configuration (p < 0.001). The detailed model is presented in Table 2. Results were similar when the third molars were excluded (Table S1).

**Table 2:** Result of tests of between-subjects effects of age, number of missing teeth (with third molars), and sex on the centroid size (CS) variables.

Dependent Variable	Parameter	β coefficient	95% CI		
			Lower Bound	Upper Bound	P value
ln(CS) cranial base	intercept	4.950	4.941	4.959	0.000
	age	0.002	0.001	0.003	0.000
	number of missing teeth	0.000	−0.001	0.001	0.839
	female (male: reference)	−0.029	−0.035	−0.023	0.000
ln(CS) maxilla	intercept	4.962	4.950	4.975	0.000
	age	0.006	0.005	0.007	0.000
	number of missing teeth	−0.003	−0.004	−0.002	0.000
	female (male: reference)	−0.027	−0.035	−0.019	0.000
ln(CS) mandible	intercept	5.254	5.241	5.268	0.000
	age	0.007	0.007	0.008	0.000
	number of missing teeth	−0.001	−0.002	0.000	0.142
	female (male: reference)	−0.041	−0.049	−0.032	0.000
ln(CS) whole facial configuration	intercept	6.200	6.189	6.210	0.000
	age	0.005	0.005	0.006	0.000
	number of missing teeth	−0.002	−0.003	−0.001	0.000
	female (male: reference)	−0.037	−0.044	−0.031	0.000

On average, females and males with tooth agenesis had respectively 0.59% and 0.56% smaller craniofacial configurations than their age- and sex-matched control groups (no tooth agenesis, without considering third molars). Similarly, the size of the maxilla was smaller in subjects with agenesis than in controls, both in females (0.50%) and males (0.52%) (Table 3). This effect increased with the number of missing teeth (Table 2 and Table S1). For example, according to the regression model presented in Table 2, a 15-year-old female with no, one, or ten missing teeth will have a maxilla with a centroid size of 152.17, 151.71, or 147.67 mm. In this case, the maxillary size reduction attributed to tooth agenesis is approximately 0.5 mm per missing tooth. Regarding the whole configuration of the same case, with no, one, or ten missing teeth the centroid size would be 511.83, 510.81, or 501.70 mm, respectively. In this case, the size reduction is approximately 1 mm per missing tooth.

**Table 3:** Centroid size (CS) variables in subjects without (control) and with at least one missing tooth (agenesis, mean: 2.7 teeth per subject), without considering third molars.

Variables		Control		Agenesis		Size difference*
		ln(CS)	CS (mm)	ln(CS)	CS (mm)	
Cranial base	females	4.9494	141.08	4.9506	141.27	ns
	males	4.9774	145.10	4.9797	145.43	ns
Maxilla	females	5.0165	150.88	5.0115	150.12	−0.504%
	males	5.0415	154.70	5.0362	153.89	−0.524%
Mandible	females	5.3152	203.40	5.3227	204.93	ns
	males	5.3543	211.51	5.3608	212.89	ns
Whole facial configuration	females	6.2349	510.27	6.2291	507.28	−0.586%
	males	6.2705	528.73	6.2649	525.77	−0.560%

*Mean size difference of the agenesis from the control groups, shown only for statistically significant results; n = 808; females: n = 238, males: n = 166 per group.

ns = non-significant.

## Discussion

Using a large sample and geometric morphometric methods to analyze craniofacial form we showed here that modern individuals with tooth agenesis have smaller facial configurations than individuals without. No effects were evident for the cranial base and the mandible. The results were robust whether including or excluding the third molars from the analysis.

The potential third molar influence on the results was explored because tooth agenesis studies tend to exclude third molars, due to their highly frequent absence^1^. The worldwide average of third molar agenesis is around 22%^28^. Agenesis of third molars is more or less considered a physiologic finding or an evolutionary adaptation of the dentition rather than a developmental anomaly^29^.

The fact that no effect was evident for midline cranial base structures confirms the hypothesis of a conserved modular structure, with reduced morphological variation in humans, which has been previously supported^30,31^. The absence of an effect in the mandible could be explained by its developmental timing, which does not correspond to the timing of tooth formation; while the latter ceases early in development, mandibular development continues until late adolescence^30^. The differences in developmental formation of the mandible, the maxilla, and the cranial base including their different growth centers and sites responding to various genetic and environmental factors, might also explain our findings. Current craniofacial development concepts describe the enlargement of the brain as the main governing force for cranial and cranial base development^32^, which cease earlier compared to that of the maxilla and the mandible^30,31^. The cranial base is known to have a central role in the growth and patterning of the skull^31,32^. The mandible has a mixed mechanism of formation, which offers increased mechanical stability, already from the early stages, through Meckel’s cartilage, and is also adaptable to mechanical stimuli. The increased functional stimuli that the mandible receives and its high adaptability to functional demands may have skewed any relation of mandibular size to tooth agenesis^33,34^. The maxilla, as well as the orbit^35^, develops mainly through intramembranous ossification and also stops growing earlier than the mandible, sharing more common developmental timing with the formation of the dentition. This may explain the significant association of tooth agenesis with maxillary size, detected in our study.

The mandible comprises a complex system which stems from multiple developmental modules through various integrating mechanisms. The modularity of the mandible as a whole is well-documented, though the exact developmental model has not yet been fully elucidated^36^. Proper maxillary development at early stages seems to depend on proper mandibular development^37,38^. Furthermore, it is evident, that even large maxillary defects, such as bilateral cleft lip and palate are compatible with life^39^, whereas large mandibular defects are not^37^. These can be additional arguments to support the absence of any effect on the mandible, in contrast to the maxilla.

The potential effect of the location of tooth agenesis on the size variables was tested in an exploratory manner through the incorporation of a dummy variable representing the location of missing teeth in the multivariate model (four levels: no agenesis, agenesis in the maxilla, in the mandible, in both jaws). Exploratory analyses of this variable were performed with and without accounting for the number of missing teeth, as well as with and without accounting for the third molars and did not show any significant effect or any important modification of the original results. For this reason, it was decided not to incorporate this variable in the final model. Thus, in case of tooth agenesis, the maxilla is smaller independently of whether the teeth are missing there or in the mandible. This is also in accordance to our claim of a broader relationship between tooth agenesis and overall craniofacial development.

Thus far no study has shown an association between tooth agenesis and the size of facial structures. This is an important finding for the human head size phenotype, which significantly supplements available knowledge obtained from genetic studies that suggest a common regulatory mechanism of dental and craniofacial development. Evolutionary data suggest that such mechanisms have evolved over time. During human evolution the size of the jaws and the face^8^, as well as the size^9^ and the number^10^ of teeth have declined in response to decreased functional needs^11^. On the contrary, the size of the human brain, along with the cranium, has steadily increased^12^. The above findings might imply that common gene regulatory mechanisms that have evolved over time continue to coordinately affect the number of teeth and craniofacial size in modern humans. To further test this hypothesis, we identified 112 subjects in our original sample (56 agenesis and 56 control samples, matched for age and sex), whose radiographs depicted the entire head. In these subjects, cranial size was captured in a blinded manner through a curve digitized on the outer surface of the cranium, as shown in Fig. 1B. Indeed, the results showed that there was no relation between the number of teeth and cranial size, but only with the facial configuration (Table S2).

The two-dimensional data used for size assessment have the inherent limitation of ignoring one dimension of space. We do not expect that the use of three-dimensional data would considerably change the present results, though this remains to be tested. Blinding during the primary digitization, regarding tooth agenesis, was not feasible, but the operator was blinded to the purpose of the study. The fact that the effects were evident for specific structures and absent for others suggest no such bias. Blinding was applied at the secondary digitization that included the cranium, when the operator was aware of the purpose of the study. Finally, chronological and not skeletal age was considered during sample selection and data analysis. The skeletal age might be expected to affect size variables due to growth stage, which might not always be in accordance with chronological age. However, in such a big sample as the one tested here, any inconsistencies are expected to be randomly distributed within the whole sample, and thus, they are not expected to significantly affect the outcomes. Furthermore, there is current literature suggesting that chronological age is also a significant indicator of skeletal maturation, not necessarily inferior to that of the cervical vertebrae maturation method that could be applied here^40^.

## Conclusions

Using a large sample of individuals with tooth agenesis and properly matched controls, we show here that modern individuals with a reduced number of formed teeth have indeed smaller facial configurations. Interestingly this effect was increasing by the number of missing teeth. The results were not affected by including the third molars in the analyses. We anticipate our findings to enrich the understanding of the evolution and development of the human head size phenotype and kindle future research in the field.

## Supplementary information

Supplementary material.

## Data Availability

All data are available in the main text or the extended data. The protocols and datasets generated and/or analyzed during the current study are available from the corresponding author on reasonable request.
