# Supplementary material for: Number of teeth is associated with facial size in humans
Source: Sci Rep. 2020 Feb 4;10:1820. doi: 10.1038/s41598-020-58565-8 (PMC7000397; doi:10.1038/s41598-020-58565-8)
Supplement: Supplementary file 1 — Supplementary material. [file 41598_2020_58565_MOESM1_ESM.docx]

**Supplementary material**

**Number of teeth is associated with facial size in humans**

Elias S. Oeschger, Georgios Kanavakis, Demetrios J. Halazonetis, Nikolaos Gkantidis

**Table S1.** Results of tests of between-subjects effects of age, number of missing teeth (without third molars), and sex on the centroid size (CS) variables.

| Dependent Variable | Parameter | β coefficient | 95% CI | | |
| --- | --- | --- | --- | --- | --- |
|  |  |  | Lower Bound | Upper Bound | P value |
| ln(CS) cranial base | intercept | 4.949 | 4.940 | 4.958 | 0.000 |
|  | age | 0.002 | 0.001 | 0.003 | 0.000 |
|  | number of missing teeth | 0.001 | -0.001 | 0.002 | 0.301 |
|  | female (male: reference) | -0.029 | -0.035 | -0.023 | 0.000 |
| ln(CS) maxilla | intercept | 4.958 | 4.946 | 4.970 | 0.000 |
|  | age | 0.006 | 0.005 | 0.007 | 0.000 |
|  | number of missing teeth | -0.003 | -0.004 | -0.001 | 0.003 |
|  | female (male: reference) | -0.027 | -0.035 | -0.019 | 0.000 |
| ln(CS) mandible | intercept | 5.252 | 5.239 | 5.265 | 0.000 |
|  | age | 0.007 | 0.007 | 0.008 | 0.000 |
|  | number of missing teeth | 0.000 | -0.001 | 0.002 | 0.729 |
|  | female (male: reference) | -0.041 | -0.049 | -0.032 | 0.000 |
| ln(CS) whole facial configuration | intercept | 6.197 | 6.187 | 6.208 | 0.000 |
|  | age | 0.005 | 0.005 | 0.006 | 0.000 |
|  | number of missing teeth | -0.001 | -0.003 | 0.000 | 0.047 |
|  | female (male: reference) | -0.037 | -0.044 | -0.031 | 0.000 |

**Table S2.** Result of tests of between-subjects effects of age, number of missing teeth (with and without third molars), and sex on the centroid size (CS) variables of the cranium and the face in a subsample, where whole head radiographs were available (n = 112).

| Dependent Variable | Parameter | β coefficient | 95% CI | | |
| --- | --- | --- | --- | --- | --- |
|  |  |  | Lower Bound | Upper Bound | P value |
| Including the third molars | | | | | |
| ln(CS) cranium | intercept | 5.894 | 5.875 | 5.914 | 0.000 |
|  | age | 0.001 | 0.000 | 0.002 | 0.092 |
|  | number of missing teeth | -0.002 | -0.004 | 0.000 | 0.058 |
|  | female (male: reference) | -0.015 | -0.027 | -0.003 | 0.013 |
| ln(CS) face | intercept | 6.155 | 6.132 | 6.178 | 0.000 |
|  | age | 0.006 | 0.004 | 0.007 | 0.000 |
|  | number of missing teeth | -0.004 | -0.006 | -0.001 | 0.004 |
|  | female (male: reference) | -0.019 | -0.034 | -0.005 | 0.010 |
| Not including the third molars | | | | | |
| ln(CS) cranium | intercept | 5.892 | 5.873 | 5.911 | 0.000 |
|  | age | 0.001 | 0.000 | 0.003 | 0.071 |
|  | number of missing teeth | -0.003 | -0.006 | 0.001 | 0.119 |
|  | female (male: reference) | -0.015 | -0.027 | -0.003 | 0.015 |
| ln(CS) face | intercept | 6.150 | 6.126 | 6.173 | 0.000 |
|  | age | 0.006 | 0.004 | 0.007 | 0.000 |
|  | number of missing teeth | -0.004 | -0.008 | 0.000 | 0.055 |
|  | female (male: reference) | -0.019 | -0.034 | -0.004 | 0.013 |
